# microRNA-375 inhibits colorectal cancer cells proliferation by downregulating JAK2/STAT3 and MAP3K8/ERK signaling pathways

**DOI:** 10.18632/oncotarget.15114

**Published:** 2017-02-06

**Authors:** Ran Wei, Qin Yang, Bing Han, Yan Li, Kun Yao, Xiuyu Yang, Zexi Chen, Shanshan Yang, Jiaqi Zhou, Meizhang Li, Haijing Yu, Min Yu, Qinghua Cui

**Affiliations:** ^1^ School of Life Science, Yunnan University, Kunming, Yunnan, 650091, P.R. China; ^2^ Key Laboratory for tumor molecular biology in Yunnan Province, Yunnan University, Kunming, Yunnan, 650091, P.R. China; ^3^ Kunming Institute of Botany, Chinese Academy of Sciences, Kunming, Yunnan, 650201, PR. China

**Keywords:** microRNA-375, colorectal cancer, JAK2/STAT3 pathway, MAP3K8/ERK pathway, ATG7

## Abstract

MicroRNA-375 is involved in many types of alimentary system cancers. Our previous studies showed that microRNA-375 was significantly down-regulated in carcinoma tissues compared with para-carcinoma tissues, which strongly indicates that microRNA-375 might suppress the occurrence and development of colorectal cancer. However, the mechanism underlying the microRNA-375 regulation in colorectal cancer remains unclear. In this study, we first sorted out *jak2*, *map3k8* and *atg7* as microRNA-375 targeted genes from multiple databases, and found that *jak2*, *map3k8* and their downstream genes *stat3* and *erk* were up-regulated in carcinoma tissues. Secondly, we over-expressed microRNA-375 in colorectal cancer cell lines (HCT116, Caco2 and HT29). Our results showed that in microRNA-375 over-expressing cells, JAK2/STAT3 and MAP3K8/ERK proteins were down-regulated, cell proliferation was inhibited, cell migration rate did not change. There was no significant difference on ATG7 expression between the control group and microRNA-375 over-expressing HT29/Caco2 cells, whereas microRNA-375 down-regulated ATG7 specifically in HCT116 cells. Finally, we demonstrated that expressing microRNA-375 suppressed tumor formation in nude mice. In conclusion, microRNA-375 might function as a tumor-repressive gene to inhibit cell proliferation, mainly through targeting both JAK2/STAT3 and MAP3K8/ERK signaling pathways in colorectal cancer. These findings suggest miR-375 as a promising diagnostic marker and a therapeutic drug for colorectal cancer.

## INTRODUCTION

Colorectal cancer (CRC), including colon cancer and rectal cancer, is one of the most common human malignant cancers and the leading cause of death worldwide. Early diagnosis and treatment can markedly raise the survival rate of CRC patients from 15% to 90% [1]. Present methods of early diagnosis have many defects, thus exploring of highly sensitive and low-price early-stage diagnostic methods has great realistic values.

MicroRNAs (miRNAs) are small, 22-nucleotide endogenous non-coding RNAs found in plants, animals and some virus. miRNAs could suppress target gene expression at post-transcriptional level by binding to the 3′UTR of target mRNAs [2]. Their expression patterns have been used to diagnose various types of cancer [3–5]. Recent studies have demonstrated that human body fluid such as serum/plasma contains numerous stable miRNAs, potentially paving the way towards a novel class of cancer biomarkers [6–8]. microRNA-375 (miR-375) located in chromosome 2 functions as a cancer suppressor, which showed significant lower levels in alimentary canal cancers, such as esophageal squamous cancer, gastric cancer, liver cancer and pancreatic cancer [9–11]. In primary hepatic cancer, over-expression of miR-375 suppresses the expression of aeg-1, induces apoptosis and inhibits migration of the liver cancer cells [11]. miR-375 can suppress cell growth by arresting cancer cells in G0 phase in pancreatic cancer [12].

Our preliminary data revealed much lower levels of miR-375 in CRC carcinoma than in para-carcinoma tissue, suggesting that miR-375 might involve in cell growth or migration in CRC. However, the mechanism underlying miR-375 regulation remains unclear. Our study aims to investigate the target genes and related signal pathways of miR-375 in CRC.

## RESULTS

### JAK2, MAP3K8 and ATG7 are predicted target genes for miR-375

**Figure 1:**
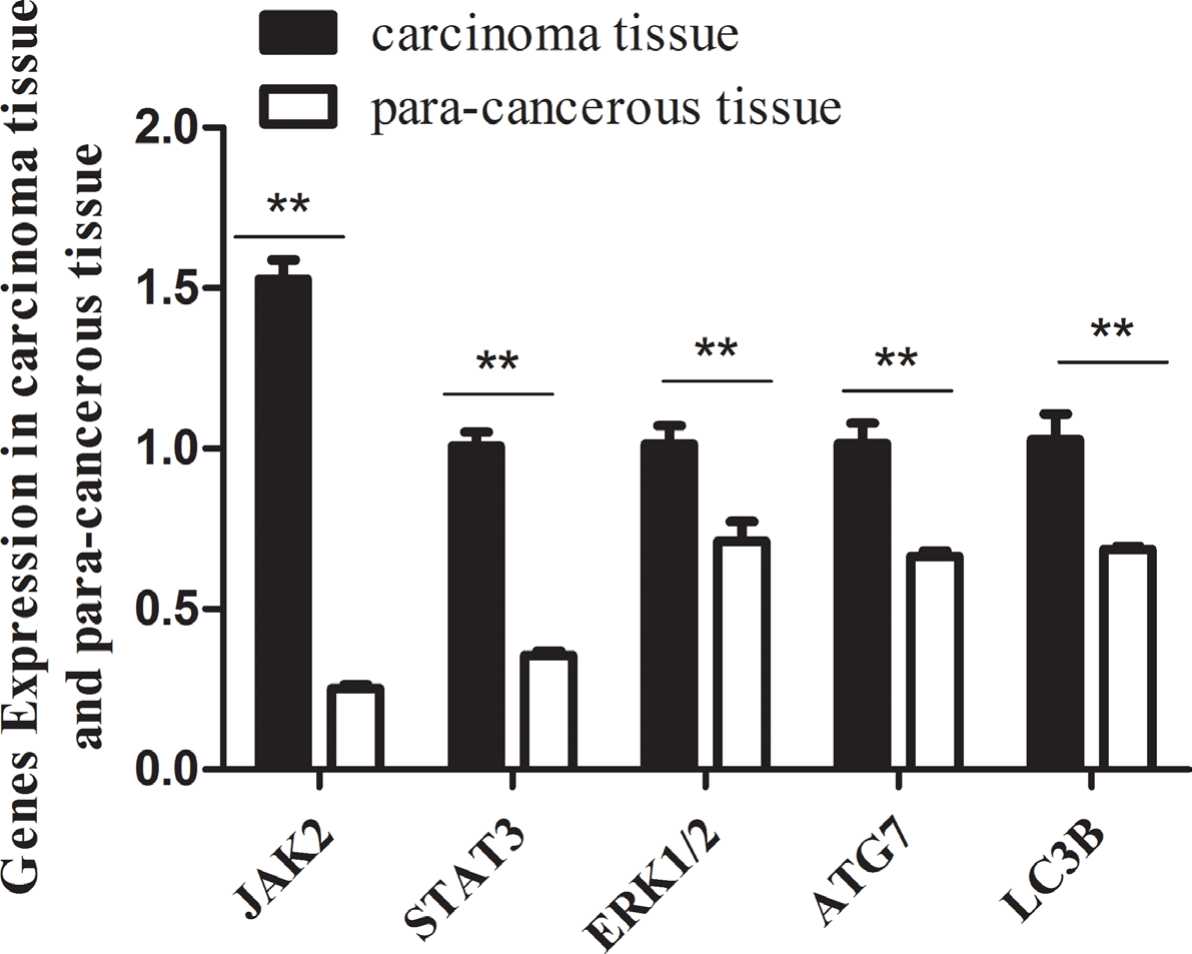
The mRNA levels of jak2, stat3, erk1/2, atg7, lc3b genes in carcinoma tissues are significantly lower than that in para-carcinoma tissues Expression level of target genes quantified by qRT-PCR (normalized by gapdh. mean ± SE are shown; *n* = 3. **p < 0.01; *p < 0.05; ns, no significance).

Our previous results showed that miR-375 has lower expression in CRC carcinoma tissues than that in para-carcinoma tissues, indicating miR-375 as a tumor suppressor. In order to test the target genes of miR-375 in CRC and the related signal pathways, we searched the databases miRTarBase, PicTar, TargetScan, miRecord and miRanda, and found that *jak2, usp1, map3k8, timm8a, yap1, pdk1, atg7* are predicted to be the target genes of miR-375. The *jak2* and *map3k8* genes belong to *jak2/stast3* and *mapk/erk* signaling pathways respectively, *atg7* relates to autophagy pathway, and these pathways were reported to modulate tumor growth or metastasis. Next, we evaluated the transcriptional levels of these predicted target genes in carcinoma and para-carcinoma tissues, the real-time PCR data showed that the mRNA levels of *jak2/stast3*, *mapk/erk* and *atg7/lc3b* genes were significantly lower in carcinoma tissues than para-carcinoma tissues (Figure 1). Thus, we speculated that miR-375 might participate in the occurrence and development of CRC by regulating these targeted genes and related signaling pathways. To explore the mechanism underlying the miR-375 regulation, we focused on the miR-375's influence on the expression of predicated genes and their downstream cascades, as well the cell proliferation and tumorigenesis in nude mice.

### Over-expression of miR-375 down-regulated the JAK2/STAT3 and MAPK/ERK signaling pathways but ATG7 down-regulation was cell line specific

**Figure 2:**
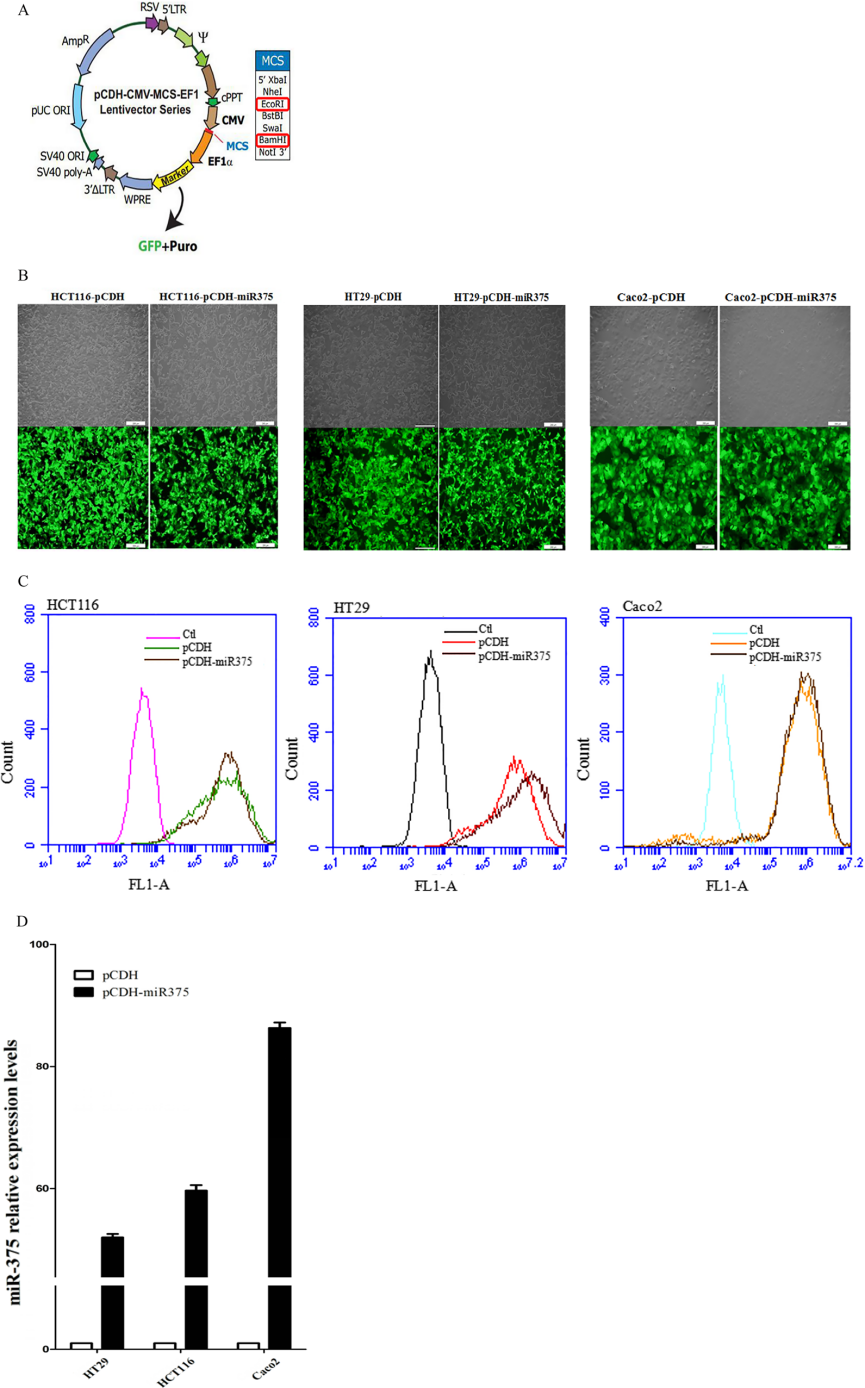
Establishment of miR-375 expressing cell lines with pCDH-CMV-MCS-EF1-GFP-T2A-Puro vector (**A**) pCDH-CMV-MCS-EF1-GFP-T2A-Puro plasmid map; (**B**) Infected cells with GFP were detected by inverted microscope. Scale bars = 100 μm. (**C**) Infection efficiency showed by flow cytometry. (**D**) Expression level of miR-375 quantified by qRT-PCR (normalized by U6. mean ± SE are shown; *n* = 3. **p < 0.01; *p < 0.05; ns, no significance).

miR-375 virus particles were produced from pCDH-miR-375 construction (Figure 2A), and infected Caco2/HCT116/HT29 cells respectively. The infection efficiency was observed by inverted microscope (Figure 2B) and detected by flow cytometry (Figure 2C). The expression level of miR-375 was quantified by Real-Time PCR (Figure 2D). miR-375 stable expressing pCDH-miR375-Caco2/HCT116/HT29 cells and pCDH vector controls were selected by puromycin.

**Figure 3:**
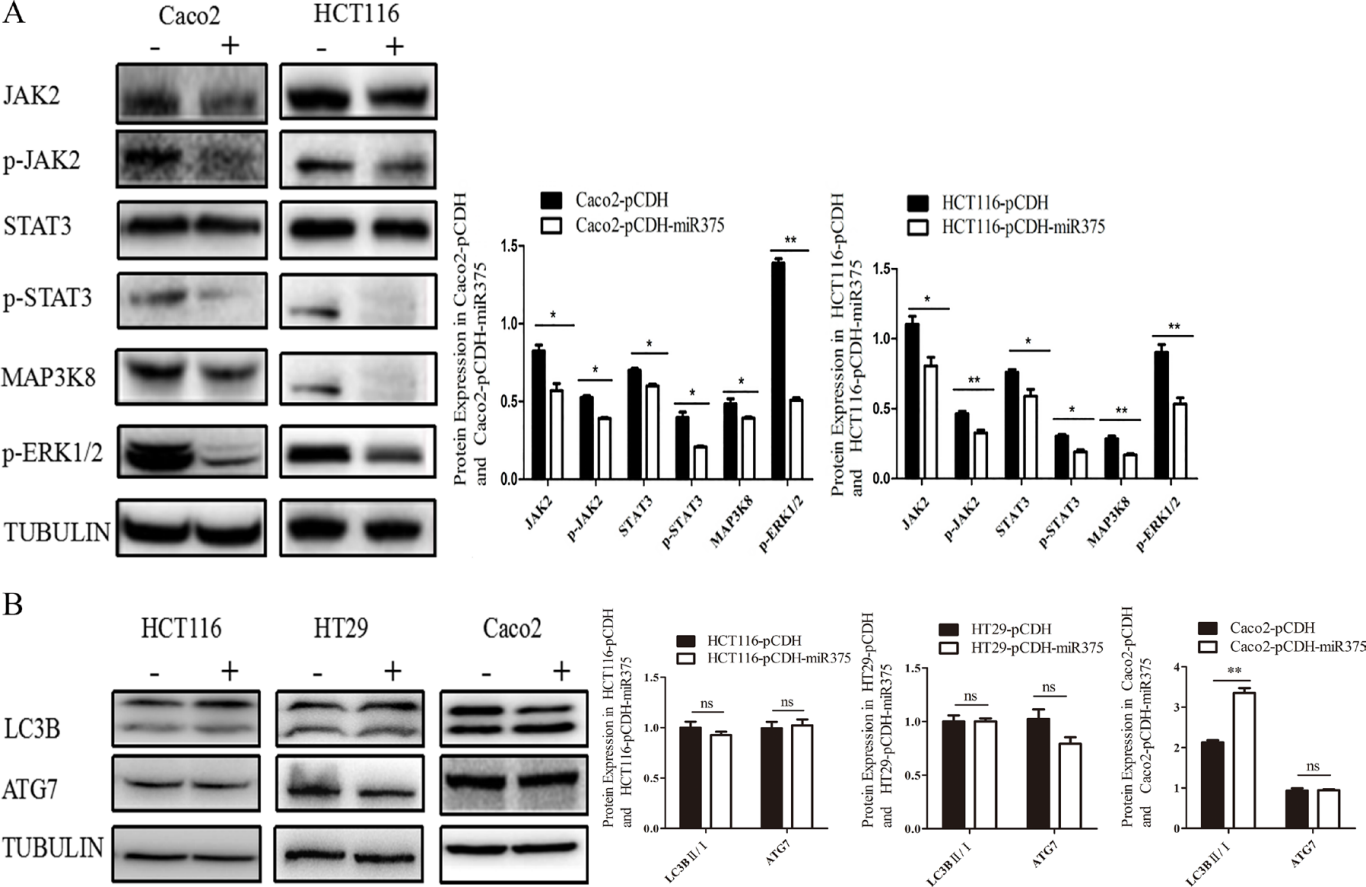
miR-375 down-regulated JAK2/STAT3 and MAPK/ERK signaling pathways, but miR-375 down-regulated ATG7 in a cell line specific way (**A**) Effects of miR375 on the protein level of JAK2/STAT3 and MAPK/ERK signaling pathways in CRC cells measured by western blot. (**B**) Effects of miR-375 on the protein level of LC3B II/I and ATG7 measured by western blot (mean ± SE of relative protein level are shown; normalized by tubulin; *n* = 3. **p < 0.01; *p < 0.05; ns, no significance).

Both group of cells were cultured for 48 h and harvested for Western Blot (Figure 3A). Our results showed that the protein level of JAK2 was down-regulated in pCDH-miR375-Caco2 and pCDH-miR375-HCT116 cells, so was the protein level of p-JAK2. The protein level of p-STAT3 in the downstream signaling pathway of p-JAK2 was down-regulated as well. The results suggest that miR-375 has inhibiting effect on JAK2/STAT3 pathway. Meanwhile, the protein level of MAP3K8 was down-regulated to a certain extent, so was the protein level of its downstream protein Erk (p-Erk). In our research, pCDH-miR375-HT29 cells showed the same trend as down-regulation of p-STAT3 and p-Erk. In conclusion, JAK2/STAT3 and MAPK/ERK signaling pathways were suppressed by miR-375.

To explore whether miR-375 also regulate autophagy in CRC, we detected another target gene of miR-375—autophagy associated gene *atg7* and LC3B-II/LC3B-I as autophagy indicator in these 3 cells lines. Western Blot results showing no significant change for ATG7 in miR-375 over-expressing HT29 and Caco2 cells, although increase of LC3B-II/I ratio indicated autophagy activation only in Caco2 cells. However, over-expression of miR-375 down-regulated ATG7 in HCT116 cells without autophagy suppression (Figure 3B). These results implied that ATG7 and autophagy might play a role in CRC, but regulated by miR-375 in a cell line specific way.

### Over-expression of miR-375 suppressed the proliferation of CRC cells

**Figure 4:**
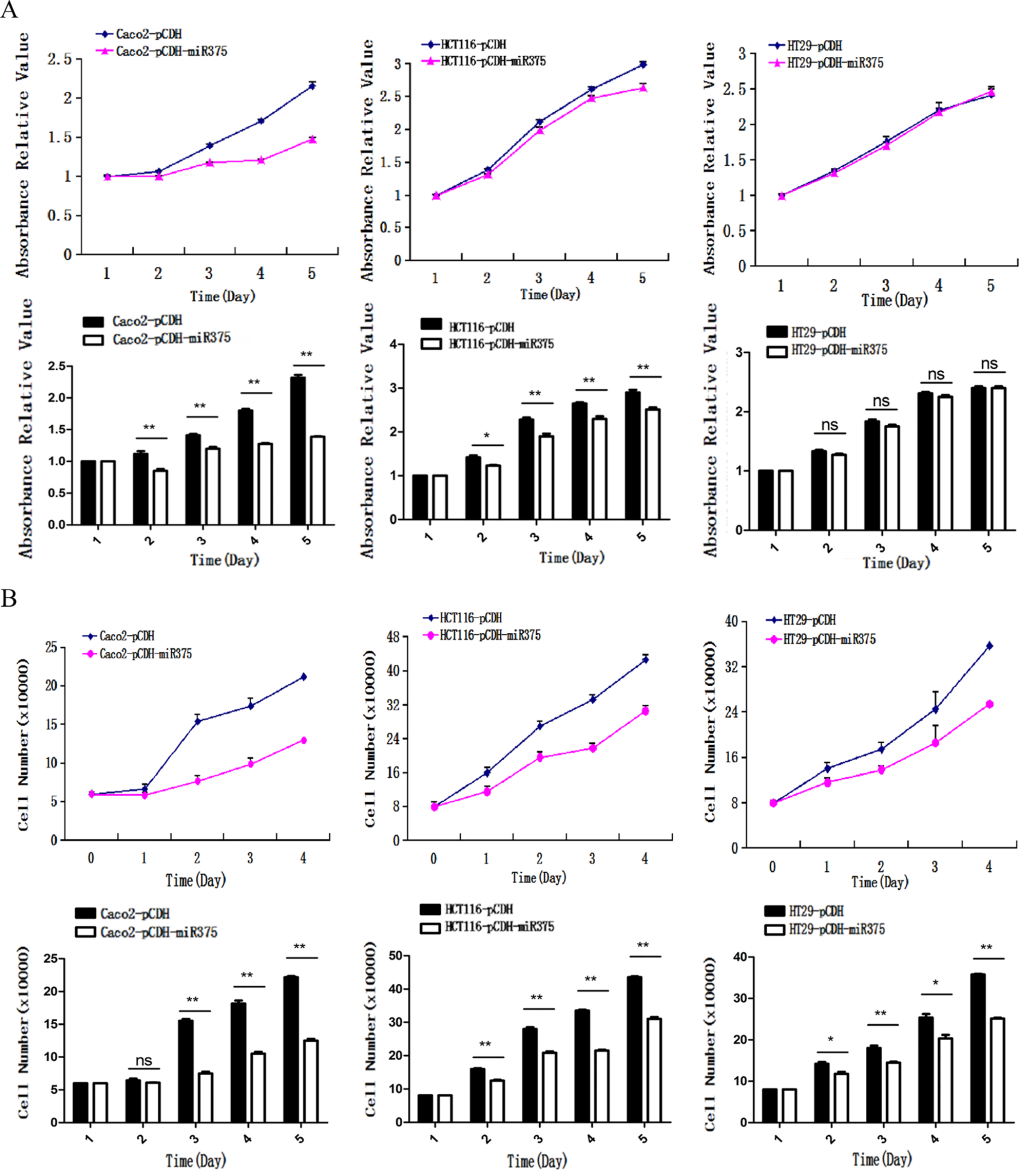
miR-375 inhibited the cell proliferation (**A**) Effects of miR-375 on the proliferation of CRC cells measured by (A). MTS assay and (**B**) cell counting assay (mean ± SE are shown; *n* = 3. **p < 0.01; *p < 0.05; ns, no significance).

Next, we explored the biological role of miR-375 at cellular level. We used MTS assay to investigate whether miR-375 was involved in the suppression of cell proliferation. A significant suppression of cell proliferation was found in pCDH-miR375-Caco2 and pCDH-miR375-HCT116 cells, though the suppression of cell proliferation in pCDH-miR375-HT29 was not significant (Figure 4A). To eliminate the deviation caused by test method, we detected the cells which were cultured at the same day by cell counting method. We found that proliferation rates of pCDH-miR375-HT29 cells, pCDH-miR375-HCT116 cells and pCDH-miR375-Caco2 cells were significantly suppressed by miR-375 since the second day of planking (Figure 4B). Collectively, these results suggest that over-expressed miR-375 has a significant inhibiting effect on the proliferation of CRC cells.

**Figure 5:**
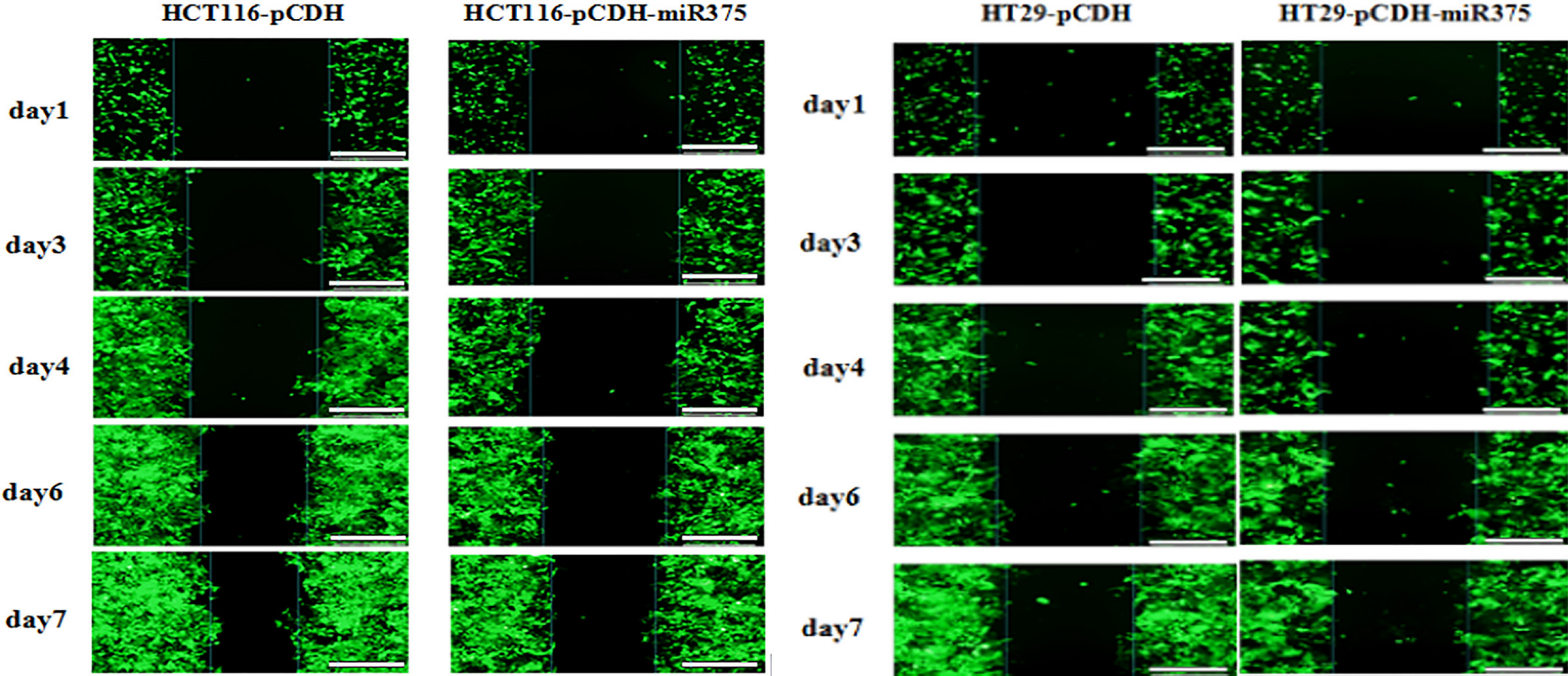
miR-375 had no effects on cell migration The cell migration for miR-375 expressing and control HT29/HCT116 cells during 7 days of wound healing assay. Scale bars = 100 μm.

In addition, we used wound healing assay to examine the effect of miR-375 on the migration of CRC cells. Caco2 cells are flat without ability of migration, so we checked HT29 and HCT116 cells only. Results showed no difference between miR-375 group and control group for healing speed (Figure 5). It suggested that miR-375 had no effect on migration in CRC cells.

### Over-expression of miR-375 suppressed the tumor formation of CRC cells in nude mice

**Figure 6:**
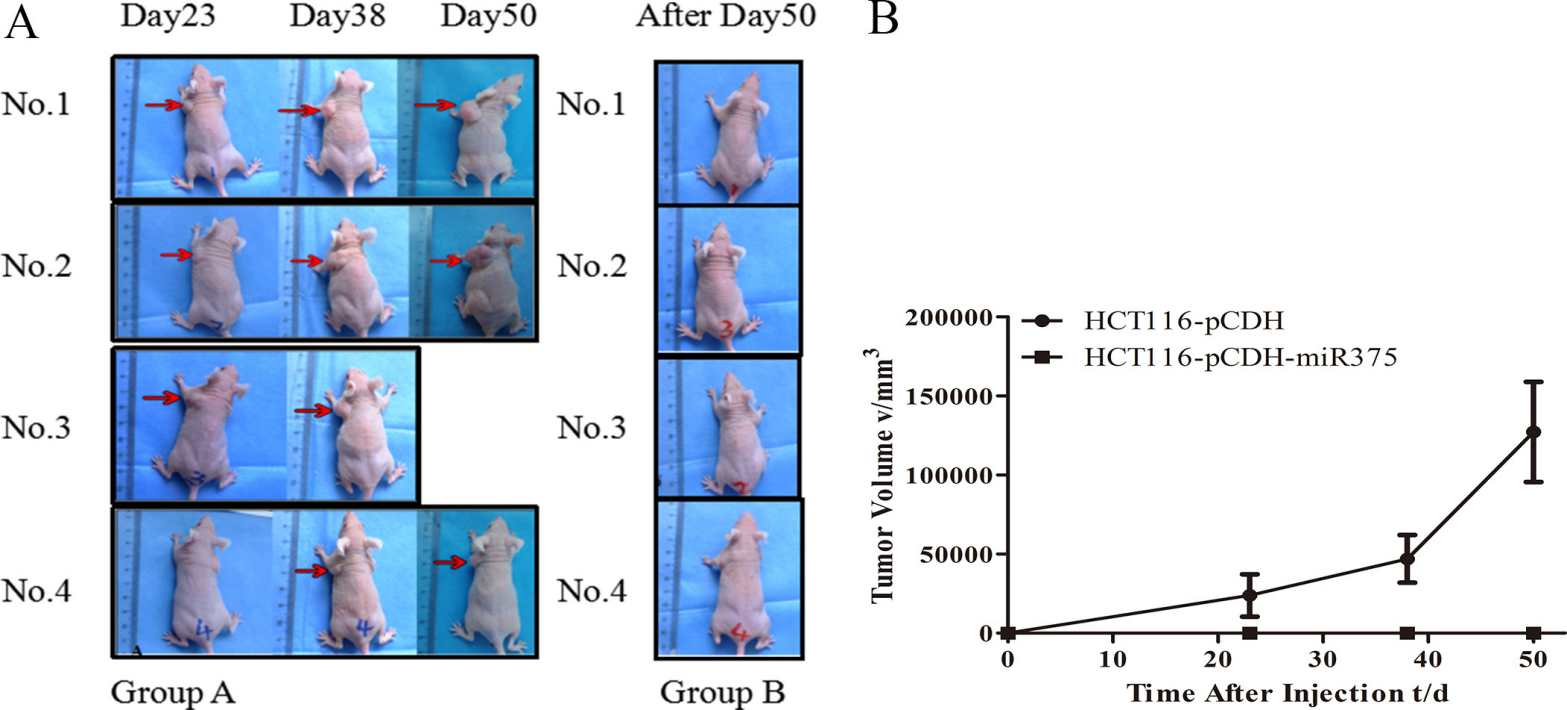
miR-375 suppressed the tumoregenesis in nude mice (**A**) Nude mice injected with pCDH-HCT116 control (Group A) and pCDH-miR375-HCT116 (Group B) (**B**) Growth curve of tumor size. (mean ± SE are shown; *n* = 4. **p < 0.01; *p < 0.05; ns, no significance).

Nude mice model was employed to identify the *in vivo* effects of miR-375 on CRC tumorigenesis. CRC bearing nude mice models were generated by subcutaneously inoculated with pCDH-miR375-HCT116 cells act as treatment group, while nude mice subcutaneously inoculated with mock-carrier (pCDH-HCT116) cells act as control group. We observed that 5 control mice appeared to form tumor at day 15 after injection, one died in day 42; while there was no tumor formation in 4 treated mice during 50-day period since injection (Figure 6A, 6B). These results indicated that the over-expression of miR-375 significantly suppressed the proliferation of CRC cells *in vivo*.

## DISCUSSION

MicroRNAs play a significant role in the diagnosis and prognosis of tumors. Hsa-miR-375 participates in the development of the pancreas through reducing the secretion of insulin in pancreatic beta cells [13, 14]. miR-375 expression suppressed the oncogene AEG-1/MTDH in head and neck squamous cell carcinoma [15], and knockdown of miR-375 increased the phosphorylation of Akt in BMPACs and post MI heart by targeting PDK-1 [16]. Recent evidence suggests an association of decreased miR-375 expression with alimentary system tumourigenesis [17]. Faltejskova [18] reported that the expression of miR-375 was significantly lower in CRC tissues than in normal colorectum tissues. In accordance with that result, our preliminary study finds that miR-375 has lower expression levels in CRC carcinoma tissues than in para-carcinoma tissues. In addition, miR-375 targeted genes (predicted by TargetScan, miRTarBase and miRecords, *jak2, map3k8* and *atg7*) were increased with their downstream genes on the mRNA level in CRC carcinoma tissues. Thus, we hypothesized that miR-375 might suppress CRC by negatively regulating these key genes. To elucidate these signaling pathways and the regulatory mechanisms of miR-375 in CRC, we constructed miR-375 stable expressing CRC cell lines, in agreement with the results in tissues, we found that *Jak2, map3k8* and their downstream genes were up-regulated in all 3 cell lines. The down-regulation of *atg7* by miR-375 showed a cell line specific manner, the autophagy levels varies in different cell lines.

The network of miRNAs, autophagy and cancer received extensive attention [19, 20]. In our study, miR-375 was significantly lower, *atg7* and autophagy (indicated by LC3II/I conversion rate) were activated in CRC carcinoma tissues. This suggests that miR-375 might mediate CRC through targeting autophagy related gene, *atg7*. However, in our cell lines, *atg7* had been silenced by miR-375 only in HCT116 cells, with no effect on the LC3II/I conversion rate. Autophagy had been increased only in miR-375 over-expressing Caco2 cells, whereas its ATG7 had no change. This implied that autophagy is a complicated process influenced by many factors, *atg7* is validated as a target gene of miR-375, but its regulation by miR-375 is cell line specific.

In our all 3 CRC cell lines, over-expressed miR-375 had an inhibited effect on both the JAK2/STAT3 and MAPK/ERK signaling pathways, leading to the inhibition of cell proliferation in CRC cells. STAT3 protein (downstream of JAK2) was found to be over-expressed and persistently activated in alimentary canal cancers, such as esophageal, stomach and colorectal cancers [21]. In gastric and liver cancers, miR-375 functions through the JAK2/STAT3 signaling pathway to control cell proliferation and apoptosis [22]. MAP3K8, a member of the Ser/Thr protein kinase family, could activate the MAP kinase and the JNK kinase pathways. The MAP3K8/ERK pathway promotes cell survival in CRC development [23, 24]. Thus, our results suggested that low expression of miR-375 activates JAK2/STAT3 and MAP3K8/ERK pathways in cancer cells, which in turn promotes cell proliferation. Furthermore, the miR-375 over-expression in nude mice significantly inhibited tumor formation without damaging the health of the mice, indicating miR-375 as a potential therapeutic target for CRC. miRNAs can be easily and sensitively detected in peripheral blood [25], which highlights miR-375 as a potential target for the non-invasive measurement for CRC.

In summary, our study observed that miR-375 suppressed the cell proliferation and tumor formation in colorectal cancer by mainly targeting both JAK2/STAT3 and MAPK/ERK signaling pathways. These findings support tumor repressive miR-375 as a potential new approach for colorectal cancer diagnosis and therapy.

## MATERIALS AND METHODS

### Ethics statement

All experimental protocols were approved by the Yunnan University Ethnics Committee, and all subjects involved in this study provided informed consent. The Institutional Animal Care and Use Committee at Yunnan University approved animal handling and procedures.

### Target prediction

The miRNA prediction was carried out using databases such as miRTarBase, PicTar, TargetScan, miRecord and miRanda.

### Cell culture

Human embryonic kidney cell line HEK293T as well as human colorectal cell line HT29, HCT116 and CaCO_2_ were cultured in DMEM (Gibco, US) supplemented with 10% fetal bovine serum (Biological Industries, IL) and 1% penicillin-streptomycin antibiotics (Hyclone, US) at 37°C in 5% humidified CO_2_ incubator.

### Vector construction

Pri-miR-375 was amplified by PCR using the sense primer 5′-GGATCCGTGTCAGCCGCAGATGCG TTCAG-3′ and anti-sense primer 5′-CTCGAGCTAGA ATCCGGGTTTCCACCTCCAG-3′. The PCR products were cloned into BamH I/EcoR I sites of a lentiviral vector pCDH-CMV-MCS-EF1-GFP-T2A-Puro (pCDH-puro, SBI, CN).

### Packaging and infection of the lentivirus

Lentiviral particles were produced by co-transfection of HEK293 cells. Cells were cultured at the density of ∼70% in 10 cm tissue culture dishes. Recombinant pCDH-puro-miR-375 or pCDH-puro empty vector with packaging plasmid PMD2. G were added into each dish to set up teatment and control group respectively, helping with x-treme GENE HP DNA Transfection Reagent (Roche, CH). The virus particles were collected 48 h after transfection, and then infected with HT29, HCT116 and CaCo2 cells respectively, supplemented with 12 μg.mL^-1^ of infection reagent polybrene. Infected cells expressing green fluorescence were detected by flow cytometry (BD, US) to evaluate the infection efficiency, and selected miR-375 stable expressing cells using puromycin.

### Quantitative real-time PCR

Total RNAs were isolated from carcinoma tissues and para-carcimona tissues to measure the expression level of target genes, or above cells to measure the expression level of miR-375. Total RNAs were reverse transcribed into cDNA using the PrimeScript reagent Kit with gDNA Eraser (TaKaRa, JP). RNA levels were quantitatively analyzed by real-time PCR on a 7300 real time PCR system (ABI, US) with the SYBR Premix Ex Taq (TaKaRa, JP). The following primers were used, Gapdh2 sense primer: 5′-AATGAAGGGGTCATTGGTGG-3′; Gapdh2 sense anti-primer:5′-AAGGTGAAGGTCGGAGTCAA-3′; Atg7 sense primer: 5′-ATGATCCCTGTAACTTAGCCCA-3′; Atg7 anti-sense primer: 5′-CACGGAAGCAAACAAC TTCAAC-3′; Erk1/2 sense primer: 5′-TCACACAGG GTTCCTGACAGA-3′; Erk1/2 anti-sense primer: 5′-ATG CAGCCTACAGACCAAATATC-3′; Jak2 sense primer: 5′-TCTGGGGAGTATGTTGCAGAA-3′; Jak2 anti-sense primer: 5′-AGACATGGTTGGGTGGATACC-3′; Lc3b sense primer: 5′-GATGTCCGACTTATTCGAGAGC-3′; Lc3b anti-sense primer: 5′-TTGAGCTGTAAGCG CCTTCTA-3′; Stat3 sense primer: 5′-ATCACGCCTTCTA CAGACTGC-3′; Stat3 anti-sense primer: 5′-CATCCTG GAGATTCTCTACCACT-3′. Hsa-miR-375 stem-loop primer: 5′-CTCAACTGGTGTCGTGGAGTCGGCAATT CAGTTGAGTCACGCGA-3′; Hsa-miR-375 sense primer: 5′-ACACTCCAGCTGGGTTTGTTCGTTCGGCTCGC-3′; Hsa-miR-375 anti-sense primer: 5′-CTCAACTGGTG TCGTGGA-3′; U6 sense primers: 5′-CTCGCTTCGGC AGCACA-3′; U6 anti-sense primer: 5′-AACGCTTCAC GAATTTGCGT-3′ [26]; Ct values were obtained and normalized to the mRNA level of housekeeping gene U6. The relative expression was calculated according to the ΔΔG method ΔΔCt = ΔCt (sample)-ΔCt(reference gene).

### Western blot

The protein concentrations in HT29, HCT116 and CaCO_2_ cell lysates were measured by BCA Protein Assay Kit (Beyotime Biotechnology, CN) according to the manufacturer's instruction. Equal amount of protein for each sample was electrophoresed on a 10% SDS-polyacrylamide gel and then transferred to a nitrocellulose membrane by electronic blotting. STAT3, p-STAT3, JAK2, p-JAK2 and p-Erk1/2(Cell Signaling, US) were used as primary antibodies. MAP3K8 (Abcam, GB), Anti-rat (Beyotime Biotechnology, CN) and anti-mouse (Beyotime Biotechnology, CN) were used as secondary antibodies. The results were visualized by chemiluminescence with Super Signal West Pico (Thermo, US). The expression level of the target protein was normalized to the expression of α-Tubulin (Beyotime Biotechnology, CN). The expression data were analyzed with ImageJ.

### Cell proliferation assay

The cell proliferation rates were evaluated by 3-(4,5-dimethylthiazol-2-yl)-5-(3-carboxymethoxyphenyl)-2-(4-sulfophenyl)-2H-tetrazolium inner salt. MTS assay was carried out at different time points (0, 24, 48, 72 and 96 h), using the Cell Titer 96 AQueous Assay (promega, USA) according to the manufacturer's instruction. pCDH-miR-375 and pCDH-puro control cells were incubated respectively in 96-well plates at the density of 4 × 10^3^ cells/well. 1× phosphate-buffered saline (PBS) was added into the fringe wells to prevent edge effects, followed by incubation for 4 h at 37°C. The absorbance of each well was measured at regular interval under 490 nm (OD_490_) using a Microplate reader (BioRad, USA), the number of viable cells were calculated and normalized to standards.

### Cell migration assay

The cell migration abilities were evaluated by wound healing method. pCDH-miR375-HCT116 cells and pCDH-miR375-HT29 cells were considered as treatment group, while pCDH-HCT116 cells and pCDH-HT29 cells were considered as control group. Both groups of cells were dispensed into 6-well plates at the density of 6 × 10^5^ cells/well with 2 mL DMEM containing 10% FBS. After 24 h incubation, a straight line of cells in each well were scraped by 200 μL pipette tips, washed 3 times by 1× phosphate-buffered saline (PBS). 2 mL culture medium was immediately added into each well and incubated for 7 days. Images of the wounded area were captured at 0, 48, 72, 120 and 144 h by inverted microscope.

### Animal protocol for miRNA injection experiment

Eight-week-old nude mice with a BALB/c genetic background were randomized into treatment and control groups (5 each), and were injected subcutaneously. Mice inoculated with 2 × 10^6^ of pCDH-miR375-HCT116 cells were considered as treatment group, while nude mice injected with same amount of mock-carrier (pCDH-HCT116) cells were considered as control group. Tumor formation and body weight were recorded for 8 weeks after injection. The volume of tumors was was calculated according to the V = 1/2*l*w*h [28].

### Statistical analysis

All data in the figures are expressed as mean ± SE (standard error of the mean) of n biological replicates. The statistical significance was valued by student t tesing (between 2 groups) or one-way ANOVA (≥ 3 groups) using graphpad prism software.
